# Life course trajectories of alcohol consumption in the United Kingdom using longitudinal data from nine cohort studies

**DOI:** 10.1186/s12916-015-0273-z

**Published:** 2015-03-06

**Authors:** Annie Britton, Yoav Ben-Shlomo, Michaela Benzeval, Diana Kuh, Steven Bell

**Affiliations:** Research Department of Epidemiology and Public Health, University College London, 1-19 Torrington Place, London, WC1E 6BT UK; School of Social & Community Medicine, University of Bristol, Canynge Hall, 39 Whatley Road, Bristol, BS8 2PS UK; Institute for Social & Economic Research, University of Essex, Wivenhoe Park, Colchester, Essex CO4 3SQ UK; Institute of Health and Wellbeing, University of Glasgow, 1 Lilybank Gardens, Glasgow, G12 8RZ UK; MRC Unit for Lifelong Health & Ageing at UCL, 33 Bedford Place, London, WC1B 5JU UK

**Keywords:** Alcohol, Life course, Longitudinal

## Abstract

**Background:**

Alcohol consumption patterns change across life and this is not fully captured in cross-sectional series data. Analysis of longitudinal data, with repeat alcohol measures, is necessary to reveal changes within the same individuals as they age. Such data are scarce and few studies are able to capture multiple decades of the life course. Therefore, we examined alcohol consumption trajectories, reporting both average weekly volume and frequency, using data from cohorts with repeated measures that cover different and overlapping periods of life.

**Methods:**

Data were from nine UK-based prospective cohorts with at least three repeated alcohol consumption measures on individuals (combined sample size of 59,397 with 174,666 alcohol observations), with data spanning from adolescence to very old age (90 years plus). Information on volume and frequency of drinking were harmonised across the cohorts. Predicted volume of alcohol by age was estimated using random effect multilevel models fitted to each cohort. Quadratic and cubic polynomial terms were used to describe non-linear age trajectories. Changes in drinking frequency by age were calculated from observed data within each cohort and then smoothed using locally weighted scatterplot smoothing. Models were fitted for men and women separately.

**Results:**

We found that, for men, mean consumption rose sharply during adolescence, peaked at around 25 years at 20 units per week, and then declined and plateaued during mid-life, before declining from around 60 years. A similar trajectory was seen for women, but with lower overall consumption (peak of around 7 to 8 units per week). Frequent drinking (daily or most days of the week) became more common during mid to older age, most notably among men, reaching above 50% of men.

**Conclusions:**

This is the first attempt to synthesise longitudinal data on alcohol consumption from several overlapping cohorts to represent the entire life course and illustrates the importance of recognising that this behaviour is dynamic. The aetiological findings from epidemiological studies using just one exposure measure of alcohol, as is typically done, should be treated with caution. Having a better understanding of how drinking changes with age may help design intervention strategies.

**Electronic supplementary material:**

The online version of this article (doi:10.1186/s12916-015-0273-z) contains supplementary material, which is available to authorized users.

## Background

Alcohol consumption and its associated harms are high on the public health agenda [1]. In the UK it is estimated that there were 8,367 alcohol-related deaths in 2012 [2] and that 8% of all hospital admissions involved an alcohol-related condition [3]. In order to identify high risk drinkers and plan for resource allocation, an accurate estimate of exposure to alcohol in the population is needed. In addition to sales data from industry sectors [4], estimates are typically drawn from cross-sectional population surveys such as, for example, the General Lifestyle Survey [5], the Health Survey for England [6], and the Scottish Health Survey [7]. Such surveys can help identify high risk groups in society [8], describe trends over time [9,10], and predict the associated burden of harm and costs [11].

Population cross-sectional surveys can also be used to compare consumption across age-groups [12]. However, cross-sectional surveys are limited as they are fixed in one specific historical moment. Alcohol consumption levels fluctuate across life [13,14] and only analysis of longitudinal data, with repeat alcohol measures, is able to reveal changes in consumption within the same individuals as they age [15]. Estimating alcohol consumption trajectories as people age and mature through the life course can ultimately be used to identify associated harm [16,17]. This allows for the investigation of whether there are sensitive periods during life when certain patterns of alcohol consumption are more harmful, and whether the impact of drinking accumulates over time [18]. Such information can be used to inform public health initiatives and sensible drinking advice.

Unfortunately, there is a paucity of datasets that are able to describe individual trajectories over the whole life course, with most focussing on adolescence to early adulthood [19-23] or mid-life into older age [24-29]. Previous attempts to synthesise data from several cohorts [30,31] in order to map across all ages are hampered by the inclusion of studies with only two time-point measurements of alcohol; this is not considered sufficient to examine trajectories [32] and may give rise to statistical issues such as regression to the mean [33]. An alternative to examining life course trajectories in a single cohort and an improvement over sequential cross-sectional analyses, is to compare data from multiple cohorts with repeated measurement that cover different and overlapping periods of life [34], as has been undertaken to examine blood pressure trajectories [35].

In this paper, we explore the extent to which alcohol consumption (mean weekly volume and drinking frequency) changes over the life course using data from nine UK-based cohorts with at least three repeated measures on individuals, with data spanning from adolescence to very old age (90 years plus). To our knowledge, this is the first attempt to synthesise information from overlapping large population-based cohorts to represent alcohol consumption across the entire life course.

## Methods

### Study populations

All cohorts included in these analyses have at least three repeat measures of alcohol consumption (volume and frequency) and are based in the UK (Table 1). Three are nationally representative birth cohorts: the Medical Research Council National Survey of Health and Development (NSHD; 1946 British Birth Cohort) [36,37], the National Child Development Survey (NCDS; 1958 British Birth Cohort) [38], and the 1970 British Birth Cohort (BCS70) [39]. The English Longitudinal Study of Ageing (ELSA) is a representative cohort of older people in England [40]. The Whitehall II study is a prospective cohort study of civil servants aged 35 to 55 years (at baseline) working in London [41]. Three cohorts are representative population samples from the West of Scotland (Twenty-07 study; T-07) [42]. These three cohorts are born 20 years apart (1930s, 1950s, and 1970s) with data collected from ages 15 to 37, 35 to 56, and 55 to 76 years. The Caerphilly Prospective Cohort Study (CaPS) is based on a population-based sample of men aged 45 to 59 years in South Wales [43].

**Table 1:** **Description of study populations**

**Study**	**T-07 1970s**	**1970 BCS70**	**1958 NCDS**	**1946 NSHD**	**T-07 1950s**	**Whitehall II**	**CaPS**	**ELSA**	**T-07 1930s**	**Total**
**Represented population**	Clydeside Glasgow, Scotland	UK	UK	UK	Clydeside, Glasgow, Scotland	Civil servants in London	South Wales	England	Clydeside, Glasgow, Scotland	––
**Total sample** ^**a**^	1,551	12,594	14,651	3,552	1,432	10,284	2,906	10,924	1,485	59,379
**Max sample male**	702	6,110	7,411	1,786	645	6,882	2,906	4,968	698	32,108
**No of alcohol observations (male)**	2,245	11,226	21,297	5,049	2,424	31,342	9,746	11,657	2,327	97,313
**Max sample female**	849	6,484	7,240	1,766	787	3,402	––	5,959	787	27,274
**No of alcohol observations (female)**	2,774	13,387	21,909	5,055	2,952	13,765	––	14,559	2,952	77,353
**No of alcohol measures**	5 vol	3 vol	4 vol	4 vol	5 vol	6 vol	5 vol	3 vol	5 vol	––
	3 freq	3 freq	4 freq			6 freq	5 freq	5 freq		
**Year(s) of birth**	1972–3	1970	1958	1946	1952–3	1930–1953	1918–1939	1908–1952	1932–3	––
**Age range**	15–37	16–38	23–59	36–64	34–60	35–83	45–83	50–90+	55–77	15–90+
**Years of data collection**	1987	1986	1981	1982	1987	1985–88	1979–83	1998–01	1987	1979
	1990–92	1996	1991	1989	1990–92	1991–93	1984–88	2002	1990/92	2010
	1995–97	2004	2004	1999	1995–97	1997–99	1989–93	2004	1995/97	
	2000–04		2008	2009	2000–04	2003–04	1993–97	2006	2000/04	
	2007–08				2007–08	2007–09	2002–04	2008	2007/08	
						2012–13		2010		

^a^Number of people with at least one measure of alcohol consumption.

Vol, volume; Freq, frequency; BCS70, British Birth Cohort; CaPS, Caerphilly Prospective Study; ELSA, English Longitudinal Study of Ageing; NCDS, National Child Development Survey; NSHD, National Survey of Health and Development; T-07, Twenty-07 study.

The cohorts contributed data from early life (age 15 years in T-07 1970s cohort) up to age 90 years and older (ELSA), with cohorts overlapping to some extent across different periods of life. Most of the data collection phases were from the mid-1980s onwards (Table 1). The combined sample size was 59,397 individuals (54% men) with 174,666 alcohol observations.

### Ethics statement

Ethical approval was given by the Central Manchester Research Ethics Committee (REC) for the latest NSHD data collection, which took place in England and Wales (07/H1008/245), and by the Scotland A REC. Each wave of the West of Scotland Twenty-07 Study was approved by the local NHS or University of Glasgow ethics committees. The University College London Medical School Committee on the ethics of human research approved the Whitehall II study. The CaPS study was reviewed by several different RECs over the past three decades, including South Glamorgan Area Health Authority, Gwent REC, and South East Wales REC. Ethical approval for ELSA and NCDS was granted by the London Multi-Centre Research Ethics Committee. Written and informed consent was obtained for every participant.

### Alcohol consumption

Mean weekly alcohol consumption was derived from each cohort and harmonised to UK units (where 1 unit equals 8 g ethanol [44]). Likewise, frequency of consumption was derived in each cohort and grouped as: “none in past year”, “monthly/special occasions”, “weekly infrequent”, and “weekly frequent” (daily or almost daily). Further details are available in Additional file 1.

### Statistical analysis

Predicted volume of alcohol consumed as a function of age was estimated using random effect multilevel models fitted to each cohort. In studies where age was consistent across individuals (birth cohorts), linear effects were assumed when only three measurement occasions were available (e.g., BCS70) while in studies with four or more measures, or a range of ages at each occasion, quadratic and cubic polynomial terms were used to describe non-linear trajectories. Covariance between the random coefficients was allowed. Models were fitted for men and women separately and estimated using a maximum likelihood algorithm. Model fit was evaluated using likelihood tests and examining changes in the Bayesian information criterion. Robust standard errors were calculated for the best fitting model. Changes in drinking frequency by age were calculated from observed data within each cohort and represented in terms of the mean percentage of individuals reporting a particular category of consumption at any given age. These observed trajectories were then smoothed using locally weighted scatterplot smoothing.

We then combined all cohorts into a single dataset and fitted a three-level multilevel model (observations nested within individuals nested in individual cohorts) to estimate volume of alcohol consumed as a function of age across the life course with adjustment for period (broadly defined using the decade in which the measurements took place). We used fractional polynomial terms [45,46] to best describe the shape of the trajectory and centred age at 40 years. We also included an interaction between age and period to examine potential differences in alcohol intake across the life course at different time periods (available in Additional file 2).

All analyses were performed using Stata version 13 [47]. As a form of sensitivity analysis we compared the estimates obtained from these models to those calculated among complete cases only and found a consistent pattern of results (available upon request) suggesting that our findings are unlikely to be biased under the assumption of missing at random.

## Results

**Figure 1:**
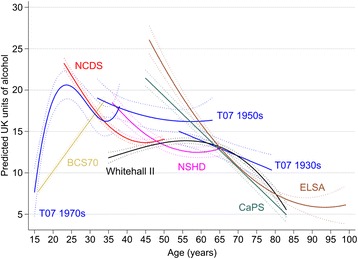
**Predicted mean alcohol consumption trajectories (in units of alcohol per week) and 95% CI across the life course in nine UK cohort studies among men.**

**Figure 2:**
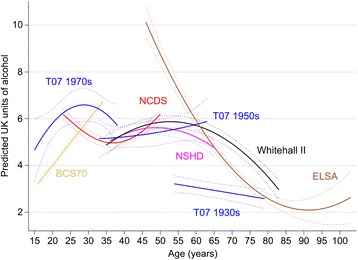
**Predicted mean alcohol consumption trajectories (in units of alcohol per week) and 95% CI across the life course in nine UK cohort studies among women.**

Among men, mean consumption rose sharply during adolescence, peaked at around age 25 years at 20 units per week, and then declined and plateaued during mid-life, before declining from around 60 years to 5 to 10 units per week (Figure 1). Similar mean trajectories were seen for women, but with lower overall consumption (peak of around 7 to 8 units per week falling to 2 to 4 units in those aged 70 and above; Figure 2). The coefficients for the age effects from these models are shown in Table 2. The steepest rise in alcohol volume was found in the Twenty-07 1970s young male adolescent cohort, where increases in three and a half units per year (standard error, 0.63) were found between ages 15 and about 25 years. The steepest decline was found among ELSA men where decreases in almost one unit per year (regression coefficient, −0.90; standard error, 0.07) were found from 45 years.

**Table 2:** **Regression coefficients (standard errors) for the fixed effects from the multilevel models displayed in Figures** 1 **and** 2

	**Study**	**Intercept age** ^**†**^	**Intercept**	**Age**	**Age** ^**2**^	**Age** ^**3**^
Men	T-07 1970s	15	7.663 (1.481)	3.525 (0.632)	−0.295 (0.065)	0.007 (0.002)
	1970 BCS	16	7.748 (0.188)	0.613 (0.018)	––	––
	1958 NCDS	23	23.222 (0.317)	−0.850 (0.046)	0.019 (0.002)	––
	1946 NSHD	36	18.467 (0.587)	−0.524 (0.062)	0.011 (0.002)	––
	T-07 1950s	33	18.804 (1.052)	−0.216 (0.158)	0.004 (0.005)	––
	Whitehall II	35	11.820 (0.338)	0.136 (0.051)	0.001 (0.002)	−0.0001 (0.00003)
	CaPS	45	21.429 (0.488)	−0.434 (0.020)	––	––
	ELSA	45	26.908 (0.926)	−0.871 (0.073)	0.009 (0.001)	––
	T-07 1930s	54	15.018 (0.794)	−0.187 (0.051)	––	––
Women	T-07 1970s	15	4.665 (0.679)	0.280 (0.132)	−0.010 (0.005)	––
	1970 BCS	16	3.235 (0.088)	0.194 (0.008)	––	––
	1958 NCDS	23	6.182 (0.113)	−0.178 (0.017)	0.007 (0.001)	––
	1946 NSHD	36	5.065 (0.214)	0.085 (0.026)	−0.003 (0.001)	––
	T-07 1950s	33	5.147 (0.407)	0.007 (0.067)	0.0006 (0.002)	––
	Whitehall II	35	4.872 (0.212)	0.113 (0.018)	−0.003 (0.0004)	––
	ELSA	45	10.481 (0.352)	−0.363 (0.027)	0.004 (0.001)	––
	T-07 1930s	54	3.215 (0.179)	−0.025 (0.009)	––	––

^†^Age in years that that age was centred in each model. BCS70, British Birth Cohort; CaPS, Caerphilly Prospective Study; ELSA, English Longitudinal Study of Ageing; NCDS, National Child Development Survey; NSHD, National Survey of Health and Development; T-07, Twenty-07 study.

**Figure 3:**
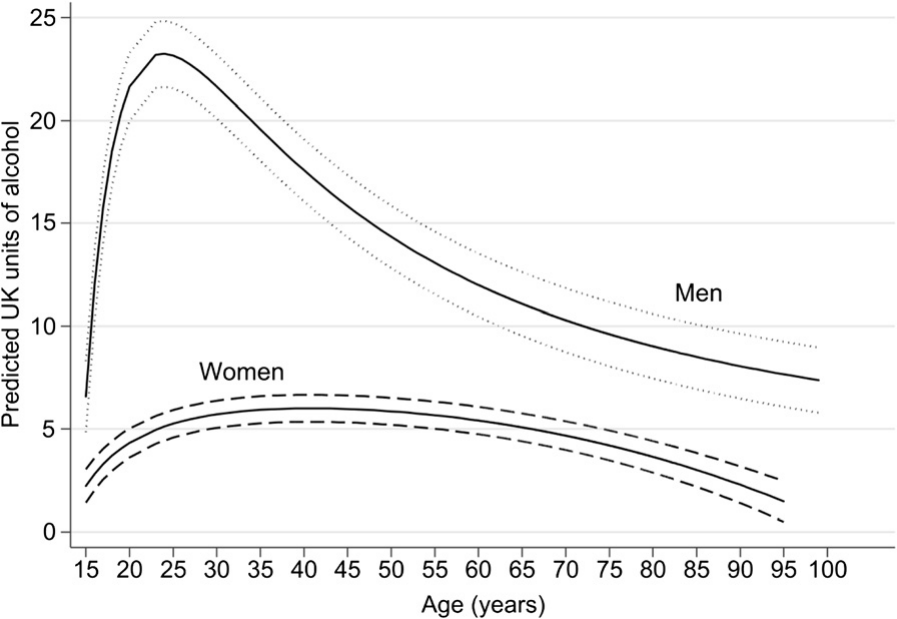
**Combined predicted mean alcohol consumption trajectories (in units of alcohol per week) and 95% CI across the life course in nine UK cohort studies among men and women.**

The combined predicted mean consumption trajectories for men and women are shown in Figure 3. These show the sharp increase in volume during adolescence followed by a gradual decline as people age.

**Figure 4:**
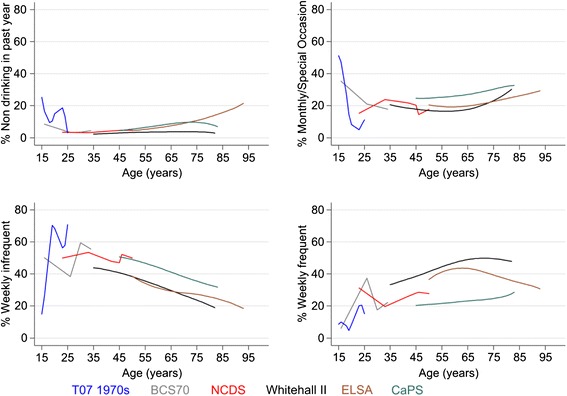
**Smoothed plots of drinking frequency by age among men in five cohorts.**

**Figure 5:**
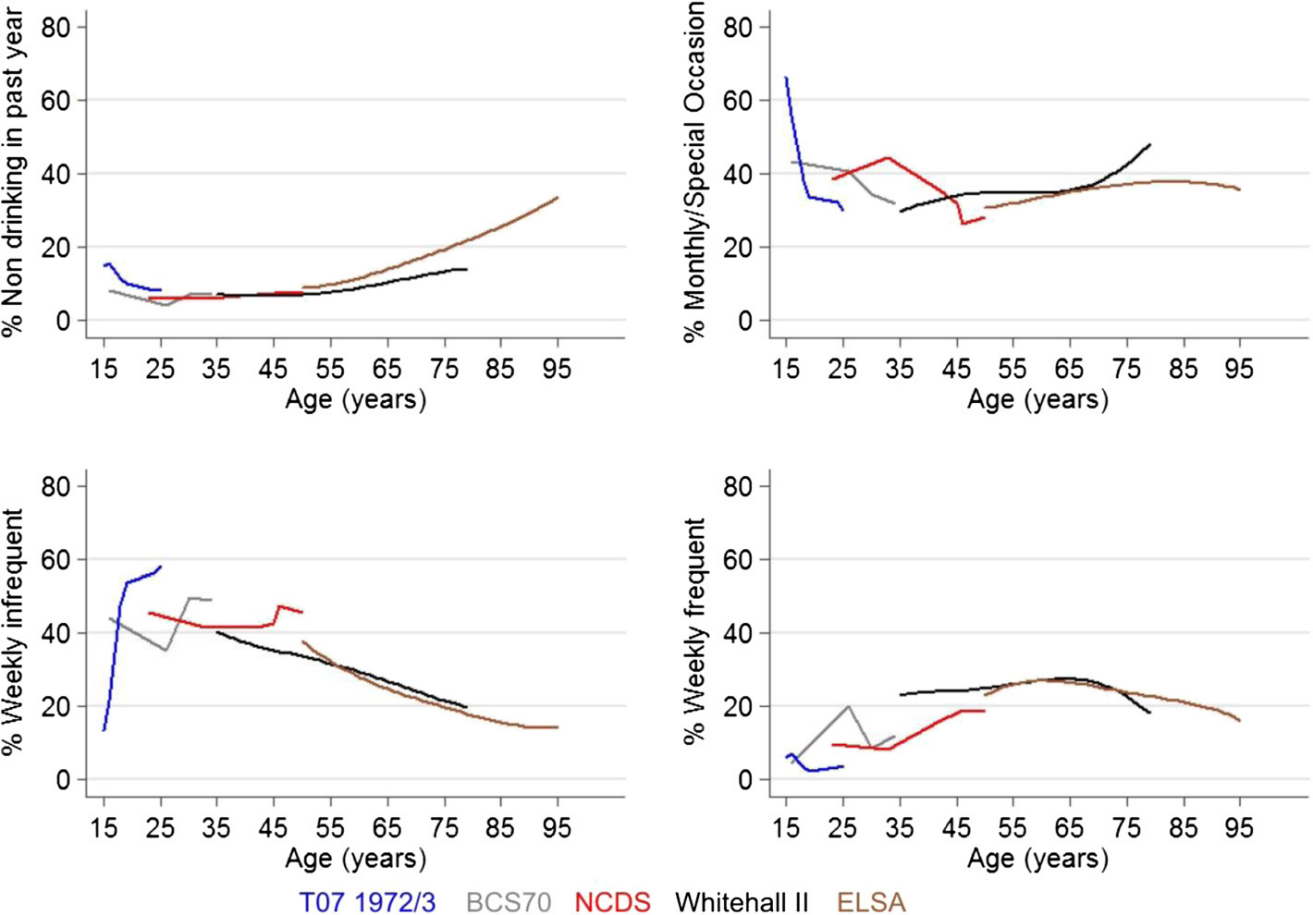
**Smoothed plots of drinking frequency by age among women in five cohorts.**

Non-drinkers were uncommon, particularly among men, where the proportion remained under 10% until old age, when it rose to above 20% among those aged over 90 (Figure 4). Drinking once or twice a week was prevalent among adolescents and those in their twenties. Drinking only monthly or on special occasions was more common among women than men (Figure 5). Frequent drinking (daily or most days of the week) became more common in middle to old age, most notably among men, reaching above 50% in men aged 65 years and over in the Whitehall II cohort. Frequent drinking declined in very old age.

Each cohort was overlapped to some extent by at least one other cohort with data at a similar age. Whilst the volume trajectories were broadly similar across cohorts at the same age points, there were some notable differences. For example, mean consumption was lower among men in the Whitehall II cohort and higher in the CaPs and ELSA cohorts at around ages 45 to 50 years. Older women in the West of Scotland cohort (T-07 1930s) reported much lower consumption than women of a similar age in the Whitehall II cohort. These data were collected at similar times (2002 onwards) so are unlikely to reflect period effects.

There were slight differences in rate of change in alcohol consumption across the life course by period; however, the overall shape of the trajectory remained the same (a peak in early adulthood followed by a decline from midlife onwards; Additional file 2).

## Discussion

This is the first attempt to synthesise and harmonise information on alcohol consumption from overlapping cohorts to represent the whole life course, using data from large population based cohorts of men and women, with multiple repeated measures of consumption as they age. Our analyses describing the average volume of alcohol consumed showed a rapid increase in consumption during adolescence, followed by a plateau in mid-life, and then a decline into older ages. Drinking occasions, on the other hand, tended to become more frequent with daily/nearly daily consumption being most common in older men, suggesting a shift away from irregular drinking in earlier years. This latter finding supports concerns raised recently about the misuse of alcohol among older people [48].

The trajectories are based on over 174,000 alcohol observations. With a minimum of three repeated measurements from each cohort, we were able to model non-linear effects. This was a serious limitation of previous work [30,31]. Great care was taken to harmonise the information on volume and frequency despite it being gathered using different questionnaires; however, we were not able with these datasets to capture full details of drinking pattern or context of drinking occasions over time. The volume and frequency estimates were reliant upon self-report, which may lead to both over- and under-estimation [49] and the strength of some alcohol consumed is likely to have increased over time [12]. Estimates of population level alcohol consumption from surveys are lower than sales data suggest, largely due to their failure to capture heavy drinking sections of society [50,51]. Furthermore, longitudinal population cohort studies are at risk of selective attrition, which may mean that heavier drinkers were more likely to drop out [52]. However, our sensitivity analyses showed that selective attrition did not seem to affect the main results.

Our findings are broadly in agreement with individual studies on drinking behaviour change which report that alcohol consumption decreases with age [13,14,24,30], with some suggesting that drinking patterns stabilise around the age of 30 [31] and in middle age [29], whilst others suggest later at age 50 years [25]. Fillmore et al. [30] combined data from 20 longitudinal studies from Europe, US, and New Zealand to look at changes in quantity of drinking and found that mean consumption declined significantly with age in men until they reached their seventies, whilst mean consumption in women decreased slightly at ages 15 to 29 and 40 to 49 years [30]. In an updated meta-analysis of these studies, Johnstone et al. [31] evaluated drinking frequency and found settled patterns after the age of 30 years following earlier marked variation. In these meta-analyses, change in consumption was assessed using only two measurements of alcohol and, therefore, the authors were unable to estimate trajectories of change with growth curve or other dynamic modelling procedures.

The data presented in this paper were collected over a 34 year period (1979 to 2013) and the participants were born in different eras (spanning 1918 to 1973), therefore, the interpretation needs to be set against period and cohort effects [53]. To a certain extent, we were able to look at period effects by examining data collected in three different decades and found only minor differences. Furthermore, this is corroborated with data from the World Health Organisation, which suggest there have been only minor differences in the estimates of alcohol per capita over the last two decades [54]. On the other hand, the overlapping cohorts provide an opportunity to compare the robustness and time-resistant nature of the trajectories. Clearly, there are some cohort differences, which are likely to be partly attributable to covariates such as socio-economic position. The lower estimates of male alcohol consumption in the Whitehall II cohort are likely due to it being a ‘white collar’ occupational cohort compared to the other population-based cohorts. We chose not to adjust for covariates, but to present the actual trajectories for each cohort separately and combined.

Reassuringly, the estimates from the nine UK cohorts of around 15 to 20 units per week for adult men are similar to the estimates obtained in the General Lifestyle Survey (GLS), which covers Great Britain (mean of 17.8 units for men aged 45 to 64 years, 2010) [55]. The female cohort estimates of 4 to 6 units are slightly lower than the GLS estimates (approximately 8 units per week across adulthood). The GLS data also suggests declines in older age groups with lower average consumption among people aged 65 and over (12.5 units for men and 4.6 for women). However, the GLS cross-sectional data is fixed at one point in time (2010 in the case here) and should not be used alone to describe age-related alcohol trajectories.

In this paper, we focused on mean trajectories of consumption, which, by necessity, masks the individual variations. Future work will classify trajectories of lifetime drinking according to profiles (for example, persistent heavy drinker, increasing drinker, sporadic drinker, etc.) using growth mixture modelling or latent class analysis [19,56,57] and, where available, the identified trajectory profiles will be analysed in relation to outcome data such as mortality and incidence of cardiovascular disease and cancer. This will allow for the investigation of whether there are sensitive periods during life when certain patterns of alcohol consumption are more harmful and whether the impact of drinking accumulates over time [58]. Such information can be used to inform public health initiatives and sensible drinking advice.

Capturing variations in drinking pattern over time has been the focus of scientific endeavour for decades [13] and it has long been known that alcohol consumption may fluctuate over the life course. However, much of the evidence linking alcohol to health outcomes relies upon evidence from prospective cohort studies in which exposure to alcohol is measured only once at baseline. It is assumed that this initial consumption level is an accurate measure of exposure throughout the study period (which may be several decades for some health outcomes). Epidemiological studies using just one exposure measure of alcohol, as is typically done, should be treated with caution. The current evidence base lacks this consideration of the complexity of lifetime consumption patterns, as well as the major predictors of change in drinking and the subsequent health risks. Research on the health consequences of alcohol needs to address the effects of changes in drinking behaviour over the life course [59].

## Conclusions

This is the first attempt to synthesise longitudinal data on alcohol consumption from several overlapping cohorts to represent the entire life course. We found that consuming alcohol is common at all ages in the UK and that individuals change their drinking pattern substantially as they age; initial increases in volume during adolescence are followed by a more stable period during mid-life before declines in volume into older age. The frequency of intake shifts from less frequent occasions to a daily/nearly daily intake being most common among elderly men.

## Additional files

Additional file 1:
**Details on the harmonisation procedure for alcohol consumption data in the nine participating cohorts.**
Additional file 2:
**Additional analyses in the combined dataset testing for potential period effects.**

## Abbreviations

BCS70: 1970 British Birth Cohort
CaPS: Caerphilly Prospective Cohort Study
ELSA: English Longitudinal Study of Ageing
GLS: General Lifestyle Survey
NCDS: National Child Development Survey
NSHD: National Survey of Health and Development
REC: Research Ethics Committee
T-07: Twenty-07 Study
