# Supplementary material for: Life course trajectories of alcohol consumption in the United Kingdom using longitudinal data from nine cohort studies
Source: BMC Med. 2015 Mar 6;13:47. doi: 10.1186/s12916-015-0273-z (PMC4351673; doi:10.1186/s12916-015-0273-z)
Supplement: Additional file 1: — Details on the harmonisation procedure for alcohol consumption data in the nine participating cohorts. [file 12916_2015_273_MOESM1_ESM.docx]

# Additional file 1

## Harmonising alcohol data in the nine participating cohorts

Below is a summary of how information on alcohol consumption was collected and derived in each cohort. We also highlight decisions made to maximise the harmony of measures across data sets.

Measures harmonised across cohorts were **number of UK units of alcohol per week** (whereby one unit is equivalent to 8g of ethanol[1, 2]) and **frequency of consumption in the past year** (categories of none, monthly/special occasions, weekly infrequent (not on a daily/almost daily basis), and weekly frequent (on a daily or almost daily basis)). In all cohorts, participants with missing information on alcohol consumption in the previous week but who had values present for frequency of consumption in the past year were assigned a zero value.

### 1970 British Birth Cohort Study (BCS70)

Study questionnaires are available [here](http://surveynet.ac.uk/index/search.aspx?collectionid=1099&resultfields=highlight%2Cpath%2Ctitle%2Cthumbnailpath%2Cpages&resultstart=1&resultsperpage=10&xslfile=style%2Fsearch.xsl&indexdirectory=E%3A\webpages\surveynet.ac.uk\SQB\QB\TabulaDX\collections\1099\index&thumbnails=true&query=%28title%3A%221970+British+Cohort+Study%22%29&doctext=&title=%221970+British+Cohort+Study%22&title_operator=AND&doctype=&doctype_operator=AND&crossnational=).

Participants were asked how frequently they had an alcoholic drink of any kind. Participants who said that they currently drank were asked to provide details of their alcohol intake during the last 7 days.

#### Volume

At age 16 years participants were asked to report the number of glasses of shandy, beer, cider, wine, sherry/fortified wine and spirits that they consumed in the previous week.

At age 26 participants were asked to report the number of glasses of shandy, beer (regular and large), cider (regular and large), wine (regular and large), spirits and sherry/fortified wine. Participants were also asked whether they consumed any other alcoholic beverage but data were not collected on the amount of non-listed beverage types. Drinks were converted into UK units using the following conversion factors: each half pint or regular size beer/cider equated to 1 UK unit, each regular size glass of wine as 1 unit and a large glass constituting 2 UK units, and single measures of spirits and fortified wine constituting 1 unit. These converted measurements were then summed to define the total weekly number of UK units consumed.

Information on alcohol intake was also ascertained when participants were aged 30 years – however, there are documented concerns regarding the accuracy of how data on beer intake were recorded by study nurses [3].

At age 34 participants were asked to report the number of glasses of beer, wine, spirits, sherry/fortified wine, and alcopops. Again, participants were also asked whether they consumed other types of alcohol but the data were not able to be used in calculations of total weekly alcohol intake. Reported drinks were converted as above with the addition that alcopops were assumed to contain 1 UK unit. Beverage specific weekly units were added together to define total weekly consumption.

#### Frequency

Participants were asked how often in the past year they had drank alcohol.

Response options at age 16 included never, special occasions only, about once a month, about once a week, 2-3 times per week, 4-5 times per week, and every day/most day. In the harmonised frequency variable never drinkers were left as they were, special occasion only and those drinking approximately once a month were combined, as were those drinking once a week with those drinking 2-3 times per week, and those drinking 4-5 times per week with those drinking every day.

Response options at age 26 included never, special occasions only, less than monthly, 1-2 times per week, 3-4 times per week, and most days. For harmonisation purposes those drinking on a special occasion basis were merged with those drinking less than monthly. Those drinking 1-4 times a week were categorised as weekly infrequency drinkers and those drinking on most days made up the weekly frequent group.

Response options at age 34 included never (ever), never (nowadays), less than monthly/special occasions only, 2-3 times per month, once a week, 2-3 times per week, and on most days. Never and never nowadays drinkers were combined, as were monthly/special occasion drinkers. Those drinking 2-3 times a week constituted the group of weekly infrequent drinkers and those drinking on most days were defined as weekly frequent drinkers.

### Caerphilly Prospective Cohort Study (CaPS)

An overview of study data is available [here](http://www.bris.ac.uk/social-community-medicine/projects/caerphilly/about/phase-variables.html).

#### Volume

Questionnaires asked participants about their frequency of drinking alcohol, and both the type (beer/cider/stout, wine, spirits/liqueurs, and sherry/port/vermouth) and amount of alcohol they usually consumed. From these questions the volume of alcohol consumed was estimated as the number of millilitres (ml) per week. To obtain UK units of alcohol ml were divided by 10.

#### Frequency

Participants were asked as part of the food frequency questionnaire “do you take some drinks containing alcohol?” at waves 1 to 3 with response options of: never, special occasions only, not every week, weekends only, weekends and occasionally in the week, most days, and every day. At waves 4 and 5 information on drinking frequency was obtained from a standalone question “do you take any drinks containing alcohol?” with the same response options.

The category of never was used to define none drinkers, those reporting special occasion only or not drinking on a weekly basis were combined to form a monthly/special occasion only category, while those reporting drinking on weekends only or weekend plus occasional midweek drinking were categorised as weekly infrequent drinkers. Participants who indicated drinking on most days or every day were combined to create a weekly frequent drinking category.

### English Longitudinal Study of Ageing (ELSA)

Study questionnaires are available [here](http://www.ifs.org.uk/ELSA/documentation).

#### Volume

At waves 0, 4, 5 and 6 of ELSA participants were asked about their alcohol consumption in the previous week.

At wave 0 this included normal (less than 6% alcohol) and strong (≥ 6% ) beer, lager, stout and cider with options of half pints (assumed to be 1 and 1.5 UK units for normal and strong beverages respectively), large cans/bottles (2 units for normal strength and 3 for strong) and small cans/bottles (1 unit for normal strength and 2 for strong). Spirits and liquors (glasses; 1 UK unit), sherry/fortified wines (glasses; 1 UK unit), wine (glasses; 1 UK unit), alcopops (small cans/bottles; 1 UK unit) and up to three other alcoholic drinks (glasses, pints, large and/or small cans/bottles – converted into units as above).

At waves 4, 5 and 6 the distinction between strength of beer and size of glass was not made, categories were: beer (including lager and cider; 1 UK unit per half pint), wine (including sherry, port and vermouth; 1 UK unit per glass), and spirits (1 UK unit per glass).

#### Frequency

Information on drinking frequency was available at waves 0-6. Participants were asked how often in the past year had they consumed an alcoholic beverage with response options: almost every day, 5-6 days a week, 3-4 days a week, 1-2 days a week, 1-2 times per month, once every couple of months, once or twice a year, not in the last 12 months.

These were recoded into none (not in the past 12 months), monthly/special occasions (1-2 times per month and once every couple of months), weekly infrequent (1-2 and 3-4 days a week), and weekly frequent (almost every day and 5-6 days a week).

### National Child and Development Study (NCDS)

Study questionnaires are available [here](http://surveynet.ac.uk/index/search.aspx?collectionid=1099&resultfields=highlight%2Cpath%2Ctitle%2Cthumbnailpath%2Cpages&resultstart=1&resultsperpage=10&xslfile=style%2Fsearch.xsl&indexdirectory=E%3A\webpages\surveynet.ac.uk\SQB\QB\TabulaDX\collections\1099\index&thumbnails=true&query=%28title%3A%22National+Child+Development+Study%22%29&doctext=&title=%22National+Child+Development+Study%22&title_operator=AND&doctype=&doctype_operator=AND&crossnational=).

#### Volume

At age 16 participants who had drank alcohol in the previous week were asked to write down the number of drinks they consumed, including the beverage type. These drinks were then converted to UK units assuming that half a pint of beer (or similar), glass of wine or measure of spirits was equal to 1 unit.

When participants were aged 23, 33, 42 and 50 they were asked to report the number of pints of beer (including lager and cider), glasses of wine, measures of spirits and sherry/fortified wines. Pints of beer were assumed to contain 2 units whilst all other beverages were assumed to contain 1 unit.

When participants were aged 46 they were asked to report the total number of units they consumed in the previous week, providing participants with conversion factors of 1 unit equating to half a pint of beer, a glass of wine or single measure of spirits/liqueur.

Given concerns about the inconsistencies in how data on beer intake were recorded by study nurses at age 42 [3] as well as changes in the format of the question at age 46 (aggregated reports of total past week drinking are believed to lead to underestimated consumption [4]) we chose not to include these measurement occasions in the present study (previous attempts to correct this issue have been demonstrated to be ineffective [5]).

#### Frequency

Participants were not directly asked about their frequency of drinking at age 16 (they were asked to report the number of weeks since their last alcoholic beverage).

At age 23 participants were asked how often they usually consumed an alcoholic drink of any kind and were provided with response options of: most days (weekly frequent drinkers in the harmonised frequency variable), 1-2 times a week (weekly infrequent), less often than weekly, special occasions (combined into the monthly/special occasion drinkers) or never.

At age 33 participants were given options of most days (weekly frequent), 1-3 times per week (weekly infrequent), 1-3 times per month, less often than monthly (combined into the monthly/special occasion drinkers), and never.

At age 42 the possible responses were: on most days (weekly frequent), 2-3 days a week, once a week (combined to form weekly infrequent), 2-3 times a month, less than monthly/special occasions (combined into the monthly/special occasion drinkers), never now a days, and never had an alcoholic drink (combined to represent the none drinking group).

At age 46 frequency categories included: on most days (weekly frequent), 2-3 days a week, once a week (combined to form weekly infrequent drinkers), 2-3 times a month, less than monthly/only on special occasions (combined into the monthly/special occasion drinking group), never nowadays and never had an alcoholic drink (making up the none drinking group).

At age 50 the response options were: on most days (weekly frequent), 2-3 days a week, once a week (combined into the weekly infrequent group), 2-3 times a month, once a month, less often than monthly/special occasions (combined to represent the monthly/special occasion drinking group), never nowadays and never had an alcoholic drink (none drinkers combined).

### MRC National Survey of Health and Development (NSHD)

Study meta-data are available [here](http://www.nshd.mrc.ac.uk/data.aspx).

#### Volume

At age 36, 43 and 53 alcohol consumption was assessed using a food diary. Participants were asked to record the amount of alcohol they consumed over five consecutive days after initially recalling the previous two days with a nurse present (five days prospective, two days retrospective). From these diaries total alcohol consumed per day (in grams per 100ml) was derived from the quantities and type of drink(s) reported under the assumption that percentage alcohol by volume (ABV) was 12% for wine, 3.5% for beer, 17.5% for sherry, and 40% for spirits. These grams per day were then summed to create a weekly number of grams of alcohol consumed and further divided by 8 to obtain UK units per week.

#### Frequency

NSHD was not eligible for harmonisation on alcohol frequency. Diaries have information on drinking in the last week – Information on drinking frequency in the past year was not asked at 36 or 43 years. At ages 53 and 60-64 participants were asked about frequency of alcohol consumption in the past year but the response options did not permit for harmonisation on account of containing too few categories: no, yes (on special occasions only), and yes (more often); this question was asked to exclude non-drinkers from questions that followed on the amount of wine, beer and spirits that had been drunk in the previous week.

### Twenty-07 (T07)

Study questionnaires are available [here](http://2007study.sphsu.mrc.ac.uk/Interview-Schedules.html).

#### Volume

Participants were asked to recall their alcohol consumption over each day in the week preceding participation for five separate beverage types: beer (including lager and cider), wine, fortified wine, spirits and ‘other’ (e.g. alcopops). Responses were expressed in UK unit equivalents (e.g. half a pint of ordinary strength beer, lager or cider, a small glass of wine or a single measure of spirits). These reported measures were then summed to calculate total weekly alcohol intake.

#### Frequency

Frequency measures were available at the first three measurement occasions for the 1970’s cohort.

At the first wave, participants were provided with response options of: once per day (weekly frequent), twice per week, once per week (weekly infrequent), once per fortnight, once per month, a few times per year (all merged to represent a group of monthly/infrequent drinkers) and not applicable (none drinkers).

At wave 2 participants were provided categories of: daily, 4-6 days per week (combined to form a weekly frequent category), 2-3 days per week, weekly (weekly infrequent), 1-2 times per month, greater than once every 6 months, once a year or less (monthly/special occasions), varies (not included; n=4).

At wave 3 the response options were: everyday, 4-6 days a week (merged into a weekly frequent category), 2-3 days a week, once a week (weekly infrequent), once or twice a month, at least once every 6 months, once a year or less (monthly/special occasion drinkers), varies (excluded; n=5).

### Whitehall II (WII)

Study questionnaires are available [here](http://www.ucl.ac.uk/whitehallII/data-sharing).

#### Volume

Participants were asked to report the number of drinks they consumed in the previous week separately for beer (pints; including lager and cider), wine (glasses; including sherry, port and vermouth) and spirits or liqueurs (measures). Drinks were converted into UK units of alcohol using a conservative estimate of one UK unit for each measure of spirits and glass of wine, and two UK units for each pint of beer. These converted measurements were then summed to define the total weekly number of UK units consumed.

#### Frequency

Participants were asked to report the frequency of their drinking over the last 12 months by selecting one of six specified options: twice a day or more, almost daily, once or twice a week, monthly, special occasions or none. Those drinking on daily basis were combined to form the category of weekly frequent. Those drinking 1-2 times per week were coded as weekly infrequency drinkers. The categories of monthly and special occasions were combined.

**References**

1. Department of Health: *Sensible Drinking: Report of an Inter-Departmental Working Group*. London: Department of Health; 1995.

2. **International Drinking Guidelines** [http://www.icap.org/Table/InternationalDrinkingGuidelines]

3. Elliott J, Dodgeon B: *A Descriptive Analysis of the Drinking Behaviour of the 1958 Cohort at Age 33 and the 1970 Cohort at Age 34*. *Working Paper*. London: Centre for Longitudinal Studies, Institute of Education, University of London; 2007:1–34. [*CLS Cohort Studies*]

4. Dawson DA: **Methodological issues in measuring alcohol use**. *Alcohol Res Health* 2003, **27**:18–29.

5. Dale CE: **A longitudinal typology of alcohol use from young to mid adulthood in the UK: evidence from the UK national child development study**. *Ph.D.* London School of Hygiene and Tropical Medicine; 2011.
