# Supplementary material for: Life course trajectories of alcohol consumption in the United Kingdom using longitudinal data from nine cohort studies
Source: BMC Med. 2015 Mar 6;13:47. doi: 10.1186/s12916-015-0273-z (PMC4351673; doi:10.1186/s12916-015-0273-z)
Supplement: Additional file 2: — Additional analyses in the combined dataset testing for potential period effects. [file 12916_2015_273_MOESM2_ESM.docx]

**Additional file 2**

Testing period effects

Using the best fitting age terms from the pooled fractional polynomial models of alcohol intake across the life course (presented in Figure 3 of the main manuscript) we explored possible period effects by including an interaction term between age and period (broadly defined by the decade that each measurement occasion occurred in) to examine potential differences in alcohol intake across the life course at different time periods for men and women separately.


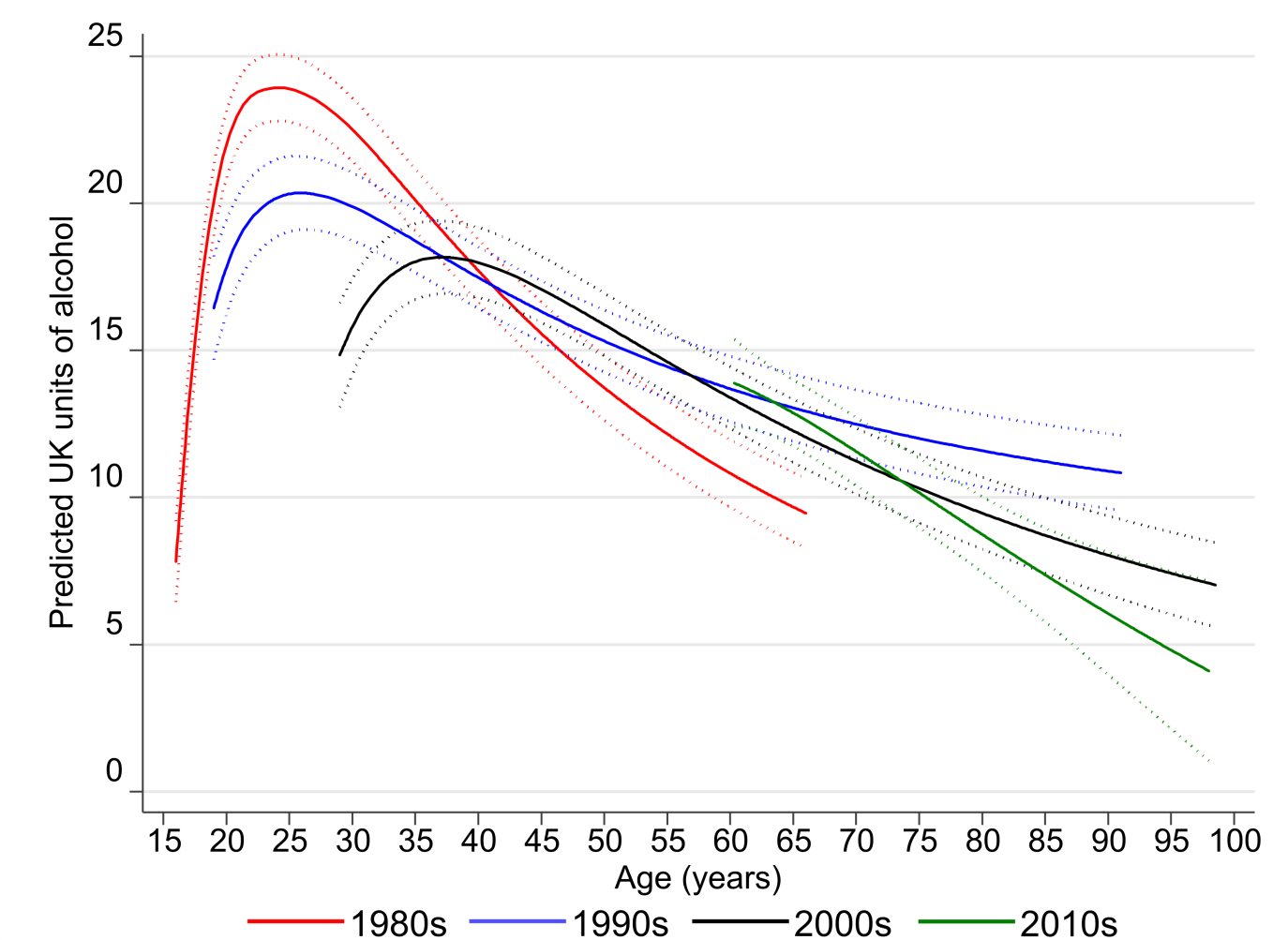


Figure 1 - Period effects of alcohol intake across the life course in men


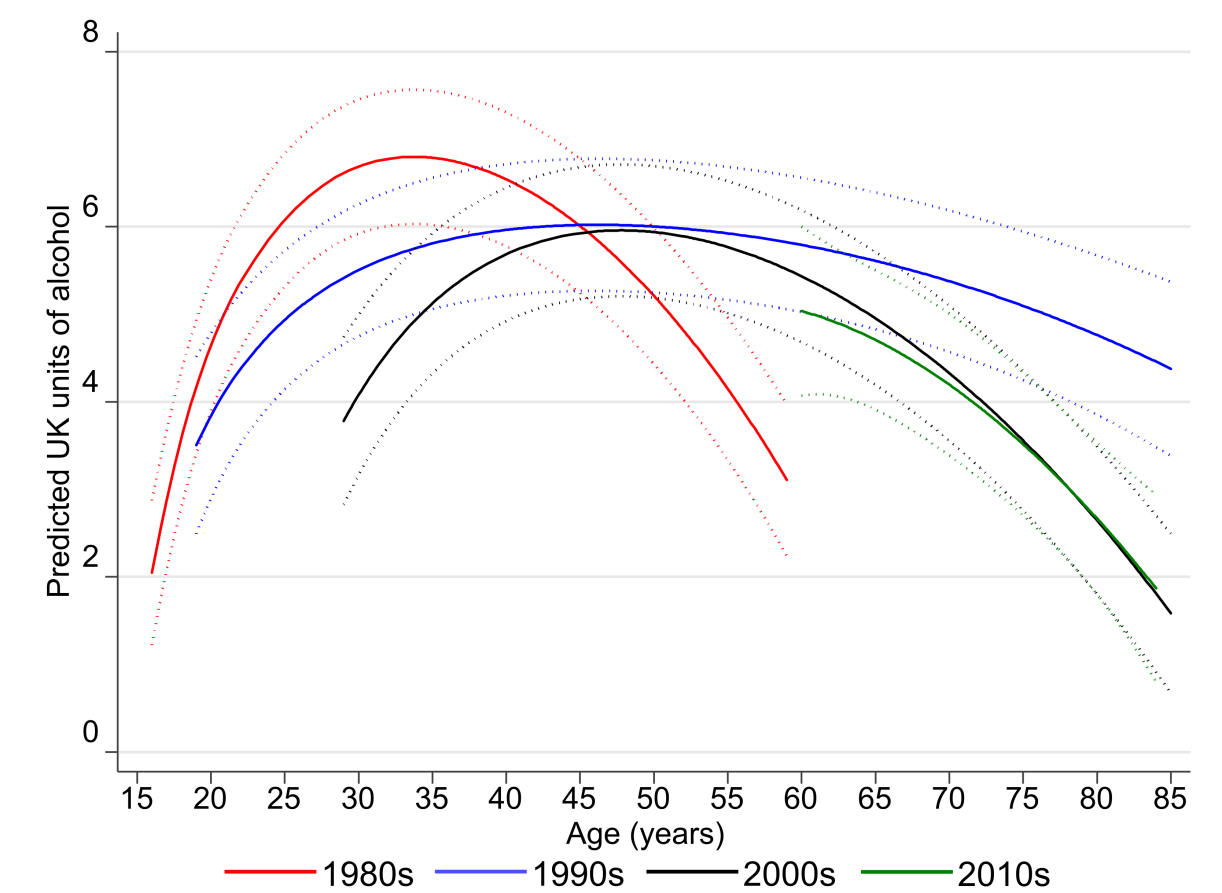


Figure 2 - Period effects of alcohol intake across the life course in women
